# Supplementary material for: Mechanisms governing the pioneering and redistribution capabilities of the non-classical pioneer PU.1
Source: Nat Commun. 2020 Jan 21;11:402. doi: 10.1038/s41467-019-13960-2 (PMC6972792; doi:10.1038/s41467-019-13960-2)
Supplement: Supplementary file 7 — Source data [file 41467_2019_13960_MOESM7_ESM.zip › Source_Data/Figure6/Figure6B_MotifScanOutput/FLI1_PU.1/homerResults/motif6.similar.html]

motif6

## Information for motif6

T
C
A
G
T
A
C
G
C
T
A
G
T
C
G
A
T
C
G
A
T
C
A
G
T
C
A
G
T
G
C
A
C
T
G
A
  
Reverse Opposite:  

G
A
C
T
A
C
G
T
A
G
T
C
A
G
T
C
A
G
C
T
A
G
C
T
G
A
T
C
A
T
G
C
A
G
T
C
  

|  |  |
| --- | --- |
| p-value: | 1e-273 |
| log p-value: | -6.295e+02 |
| Information Content per bp: | 1.679 |
| Number of Target Sequences with motif | 1348.0 |
| Percentage of Target Sequences with motif | 13.55% |
| Number of Background Sequences with motif | 1244.6 |
| Percentage of Background Sequences with motif | 3.41% |
| Average Position of motif in Targets | 97.4 +/- 56.6bp |
| Average Position of motif in Background | 105.7 +/- 86.6bp |
| Strand Bias (log2 ratio + to - strand density) | -0.3 |
| Multiplicity (# of sites on avg that occur together) | 1.03 |
| Motif File: | file (matrix) reverse opposite |

### Similar de novo motifs found

|  |  |  |  |  |  |  |  |
| --- | --- | --- | --- | --- | --- | --- | --- |
| Rank | Match Score | Redundant Motif | P-value | log P-value | % of Targets | % of Background | Motif file |
| 1 | 0.757 | C T G A C T G A A C T G G T C A C T G A T A C G T C G A G T C A | 1e-173 | -399.324956 | 24.72% | 12.72% | motif file (matrix) |
| 2 | 0.837 | A C T G C T A G A T C G A C T G C T A G C T G A A C T G A C T G C G T A C T G A | 1e-171 | -394.424727 | 16.60% | 6.93% | motif file (matrix) |
| 3 | 0.784 | T C G A T C G A C A T G A C T G T G C A C G T A A C T G T A C G G T C A G C T A A C T G T C A G G T C A C T G A | 1e-68 | -158.507323 | 5.71% | 2.10% | motif file (matrix) |
